# Supplementary material for: A water-soluble copolymer for storage and electron conversion in photocatalytic on-demand hydrogen evolution
Source: Nat Commun. 2026 Jan 28;17:1141. doi: 10.1038/s41467-026-68342-2 (PMC12855798; doi:10.1038/s41467-026-68342-2)
Supplement: Supplementary file 1 — Supplementary Information [file 41467_2026_68342_MOESM1_ESM.pdf]

# Supporting Information

## A Water-soluble Copolymer for Storage and Electron Conversion in Photocatalytic On-demand Hydrogen Evolution

*Marco Hartkorn[a], Robin Kampes[b,c], Felix Müller[a], Linda Zedler[d,e], Akuila Edwards[d], Philip Rohland[b,c,d], Alexander K. Mengele[a], Stefan Zechel[b,c], Martin D. Hager[b,c,f,g], Benjamin Dietzek-Ivanšić[d,e,h], Michael Schmitt[d], Jürgen Popp[d,e], Ulrich S. Schubert[b,c,f,g,i]*, Sven Rau[a,*]*

[a] Institute of Inorganic Chemistry I, Materials and Catalysis, Ulm University, Albert-Einstein-Allee 11, 89081 Ulm, Germany

[b] Laboratory of Organic and Macromolecular Chemistry (IOMC), Friedrich Schiller University Jena, Humboldtstraße 10, 07743 Jena, Germany

[c] Jena Center for Soft Matter (JCSM), Friedrich Schiller University Jena, Philosophenweg 7, 07743 Jena, Germany

[d] Institute of Physical Chemistry (IPC) and Abbe Center of Photonics (ACP) Friedrich-Schiller-University Jena, Helmholtzweg 4, 07743 Jena, Germany

[e] Leibniz Institute of Photonic Technology (IPHT) Jena, Albert-Einstein-Straße 9, 07745 Jena, Germany

[f] Center for Energy and Environmental Chemistry Jena (CEEC Jena), Friedrich Schiller University Jena, Philosophenweg 7a, 07743 Jena, Germany

[g] Helmholtz Institute for Polymers in Energy Applications Jena (HIPOLE Jena), Lessingstraße 12-14, 07743, Jena, Germany

[h] current address: Leibniz Institute of Surface Engineering (IOM) Leipzig

[i] Helmholtz Zentrum für Materialien und Energie Berlin (HZB), Hahn-Meitner-Platz 1, 14109 Berlin, Germany

## 1. General remarks

The catalyst [(tbbpy)Rh(Cp*)Cl]Cl and [(dceb)Rh(Cp*)Cl]Cl was synthesized according to literature.[1] The ruthenium precursor [$RuCl_2$(DMSO)$_4$] for the respective [Ru(tbbpy)$_3$]$Cl_2$ photosensitizer (PS) was synthesized according to literature.[2] The redox-active polymer was synthesized according to literature procedures.[3] The solvents used for synthetic purposes were of technical grade. 4,4'-Di-*tert*-butyl-2,2'-bipyridyl, 4,4´-Di(carboxyethyl)-2,2´-bipyridine as well as $K_2$[$PtCl_4$] and the Pt-NP-solution was obtained from Sigma Aldrich, whereas the Pentamethylcyclopentadienylrhodium(III)-chlorid-dimer was obtained from abcr. Size exclusion chromatography was performed on a Sephadex LH-20 column using MeOH as mobile phase.

<u>Synthesis of [(dceb)Rh(Cp*)Cl]Cl</u>

The catalyst was synthesized by charging a GC-vial (4.5 mL glass screw cap vials, VWR) with 11.6 mg 4,4´-Di(carboxyethyl)-2,2´-bipyridine (dceb, 38.63 µmol, 2 eq.) together with [Rh(Cp*)$Cl_2$]$_2$ (19.25 µmol, 1 eq.) dissolved in of MeOH (1 mL). The reaction mixture was stirred at ambient temperature for 2 h, before the product was precipitated by adding diethyl ether. The product was filtered off and dried to yield 2.55 mg of the pure catalyst (4.43 µmol, 11.5%).

$^1$H-NMR (400 MHz, MeOD): *δ* (ppm) = 9.18 (d, J = 5.7 Hz, 2H), 9.07 (s, 2H), 8.36 (d, J = 5.9 Hz, 2H), 4.55 (q, J = 7.2 Hz, 4H), 1.76 (s, 15H), 1.49 (t, J = 7.4 Hz, 6H).

<u>Synthesis of [(tbbpy)Rh(Cp*)Cl]Cl</u>

In a 25 mL round-bottom flask 32 mg of 4,4'-di-*tert*-butyl-2,2'-bipyridine (0.12 mmol, 2 eq.) and 37 mg of [$RhCl_2$(Cp*)]$_2$ were dissolved in 5 mL of DCM. The reaction mixture was stirred at room temperature for 4 h, before the solvent was removed. The crude product was then again taken up in small amounts of DCM and $Et_2O$ was added, resulting in the precipitation of a yellow solid. After filtering off this solid and drying it under vacuum, 69 mg (0.12 mmol 100%) of pure product were obtained.

$^1$H-NMR (400 MHz, $CDCl_3$): *δ* (ppm) = 8.79 (d, J = 5.9 Hz, 2H), 8.32 (d, J = 1.4 Hz, 2H), 7.78 (dd, J = 5.9, 1.4 Hz, 2H), 1.74 (s, 15H), 1.43 (s, 18H).

Synthesis of [$RuCl_2(DMSO)_4$]

In a 100 mL round-bottom flask 1.97 g of RuCl3 · $xH_2O$ (9.50 mmol) was taken up in 30 mL of DMSO and heated to 180 °C for 40 min. After the reaction mixture was cooled to room temperature and added to 300 mL of acetone. Storing this at 4 °C yielded multiple fractions of clean product precipitating as yellow crystals, yielding a total of 2.67 g (5.51 mmol, 58%).

Synthesis of [$Ru(tbbpy)_3$]$Cl_2$

In a 100 mL round-bottom flask 52.84 mg of [$RuCl_2(DMSO)_4$] (0.11 mmol, 1 eq.) and 87.81 mg of 4,4'-di-*tert*-butyl-2,2'-bipyridine (0.33 mmol, 3 eq.) were dissolved in a mixture of EtOH (30 mL) and water (10 mL). After degassing the solution with argon, the mixture was stirred at 85 °C for an hour. The crude product was precipitated by adding diethyl ether before further purification was performed by Sephadex column chromatography with MeOH as eluent. This way 4.69 mg of pure product (4.86 µmol, 4.5%) were obtained.

$^1$H-NMR (400 MHz, MeOD): $\delta$ (ppm) = 8.74 (d, J = 2.1 Hz, 1H), 7.68 (d, J = 6.0 Hz, 1H), 7.55 (dd, J = 6.0, 2.0 Hz, 1H), 1.47 (d, J = 1.9 Hz, 9H).

Synthesis of the viologen copolymer

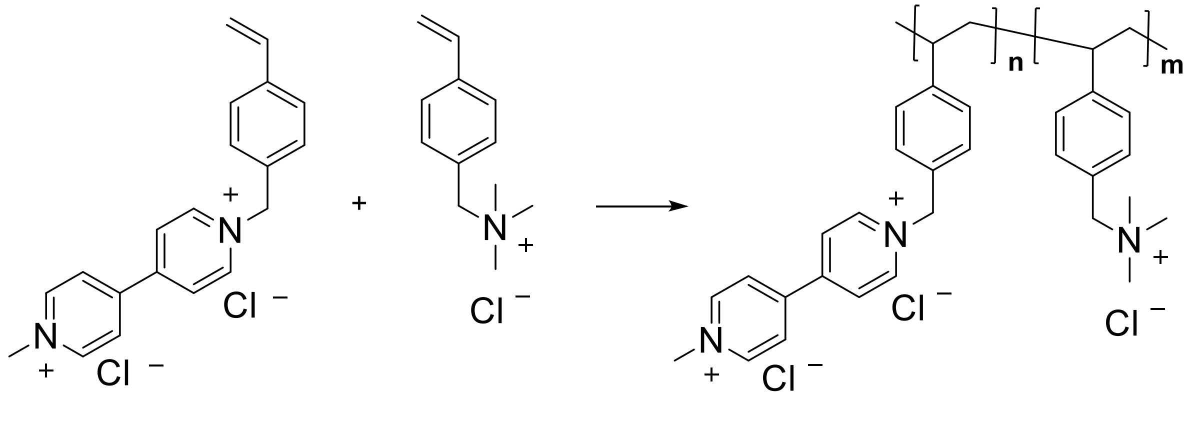


The polymer was synthesized according to literature procedures.[3] 1-Methyl-1'-(4-vinylbenzyl)-4,4'-bipyridinium chloride (5.0 g, 14 mmol) and (vinylbenzyl)-trimethyl-ammoniumchlorid (2.95 g, 14 mmol) were dissolved in 11 mL deionized water. After complete dissolution of the starting material (the dissolution can be exaggerated by

ultrasonic treatment), argon was bubbled through the solution for 10 min and the complete reaction was carried out in an argon atmosphere. Afterwards, thioglycolic acid (194 µL, 257 mg, 2.8 mmol) was added to the solution. The reaction mixture was then placed in an oil bath which was tempered to 80 °C and hydrogen peroxide (30%, 315 mg, 284 µL, 2.8 mmol) was added. After 4 h at 80 °C the mixture was cooled. The desired product was received as brownish solid (7.9 g, quant.) after freeze drying.
$^{1}$H-NMR ($D_2O$, 300 MHz): $\delta$ (ppm) = 8.98 (CH-C*H*-$N^+$), 8.48 (C*H*-CH-$N^+$),6.64 and 7.31 ($CH_{aromat.}$), 5.82 ($N^+_{aromat}$-$CH_2$), 4.42 ($N^+_{aromat.}$-$CH_3$), 2.95 ($N(CH_3)_3$), 1.57 (backbone) . From the NMR spectrum, we calculated n/m ratio of 1/1.

Absorption spectroscopy and gas-chromatography:

UV/Vis-absorption spectroscopy was performed on a JASCO V-670 UV-vis-NIR Spectrometer or an Avantes AvaSpec-ULS2048CL detector unit coupled with an AVA AvaLight-DH-S-BAL light source, using gas-tight quartz cuvettes (d = 10.0 mm, Hellma).
The amount of generated hydrogen/methane/carbon monoxide was determined by gas chromatography (GC) on a Shimadzu GC-2030 with a barrier ionization discharge (BID-2030) detector and helium as carrier gas (column: Restek SH-Rt-Msieve 5A, ID: 0.32 mm; film thickness: 30 [micro]m, length: 30 m, oven temp. 80 °C, [check current method to be sure]) using 100 µL of the gas phase. The GC was calibrated by injection of different volumes of a test gas mixture containing known percentages of hydrogen/methane/CO.

Statistics and Reproducibility

Error margins featured on the respective experiments represent the standard deviation, unless stated otherwise. These result from either triplicates, or two independent duplicates. The only data excluded in these cases is data acquired during optimization of the respective experiments, or in clear cases of an experimental error. No statistical method was used to predetermine the sample size. The experiments were not randomized. The experimenters were not blinded to allocation during experiments and outcome assessment.

Determination of the absorption characteristics of the fully reduced polymer:

All samples were prepared in an argon-filled glovebox. To be able to determine the degree of polymer charging during the catalytic measurements, several solutions of fully reduced polymer were prepared. Initially this was done by preparing 20 µM, 40 µM and 60 µM polymer solutions (concentrations are given with respect to MV-units) with three equivalents of [$CoCp^*_2$] and measuring the absorption. Later this reducing agent was replaced with the more adequate reducing agent $Na_2S_2O_4$, possessing higher solubility and stability in water.[4] With 60 equivalents of dithionite per $MV^{2+}$ unit, measurements of polymer solutions with concentrations ranging from 20 to 120 µM (in 20 µM steps) were performed. The respective absorption maxima enabled a linear fit, which allows the determination of the absorbances of a fully reduced polymer at any concentration. Inversely, the comparison of the absorbance of any samples with a known concentration to this reference enables the determination of the state of charging of the sample at any given time.

Preparation of solutions for photocatalytic charging and discharging of the polymer as well as performance of polymer charging and polymer discharging:

All following sample preparations were performed in a similar fashion. First, a solution of 900 µM copolymer solution (based on repetition units, i.e. 450 µM MV-units) containing 0.15 M of $NaH_2PO_4$ was prepared. Of this solution, 2 mL were added to 0.05 mg the photosensitizer [$Ru(tbbpy)_3$]$Cl_2$, resulting in a photosensitizer concentration of 25 µM in the utilized GC-vials (4.5 mL glass screw cap vials with septum, VWR). After addition of 50 µL of the sacrificial agent TEA to reach a concentration of 0.18 M, the samples were irradiated from the bottom of the GC-vials for one hour, utilizing a custom-made irradiation apparatus[5] equipped with one LED-stick ($\lambda_{max}$ = 465 nm, 45 to 50 mW $cm^{-2}$). Later experiments were prepared at half of the concentration of all ingredients, which is indicated respectively.
The standard discharging procedure was induced by addition of 220 µL of aqueous HCl (2 M), calculated and tested in a dummy system to yield a pH of 2, followed by the addition of the respective hydrogen evolution catalyst (HEC, $K_2PtCl_4$ (**1**), Pt-NPs (**2**), [(dceb)Rh(Cp*)Cl]Cl (**3**) and [(tbbpy)Rh(Cp*)Cl]Cl (**4**)). The concentration of the catalyst hereby was set to 20 mol-% with regard to the methyl viologen-units of the polymer (i.e. 90 µM catalyst). The initial dummy system, confirming calculations, refers

to an experiment, employing the buffer solution and the addition of respective amounts of acid and base. Depending on the measurement, either the GC-Vial (for hydrogen detection) or the inert cuvette (for UV/vis absorption spectroscopy) were sealed immediately afterwards, ready to be removed from the glovebox. Measurements without acidification were also performed (Supplementary Fig. 6) by skipping the addition of the HCl, maintaining the remaining procedure.

In a revised procedure, the dummy system relied on actual samples, rather than an upscaled titration with the buffer solution. To measure the pH-value of the actual sample a needle-pH-meter was employed, capable of piercing the septum of an inertly prepared and gastight sample vial. This method is as close as possible to the experimental conditions, which greatly enhances the precision and control over the pH-value, in particular when moving to higher cycle numbers in the multicycle experiments.

The discharging process, once it was initiated, could be observed with UV/vis absorption spectroscopy, while the catalytic activity was monitored by analyzing gas samples from the headspace and measuring the $H_2$ concentration with gas chromatography. These measurements were carried out over a time span of up to six hours in fixed, reasonable intervals to ensure comparability among various experiments. Based on these results, catalytic analysis of a cycle beyond the first one, measurements were performed after two hours and after three hours.

To enable quantitative measurement of the degree of polymer charging, a chemical reduction provided a fully reduced species as reference. Linear regression based on spectra at multiple concentrations allows the determination of the percentage to which the polymer is charged at a given concentration (see experimental description above).

<u>$pK_a$ determination of Rh hydrides (similar to literature[6]):</u>

Determination of the $pK_a$ value of two electron reduced and protonated [(dceb)Rh(Cp*)Cl]Cl: Samples of a 25 µM [(dceb)Rh(Cp*)Cl]Cl and 20 mM formate solution at different pH values were prepared, by adjusting the $NaH_2PO_4$ buffer and HCl concentration. The samples were heated to 40 °C for about 2 h before the absorption spectra were measured. A sigmoidal fit on the characteristic absorption band at 528 nm of [(dceb)Rh(Cp*)] revealed a $pK_a$ of 3.8 for the two electron reduced and protonated catalyst (see Supplementary Fig. 12).

Stability studies of [(dceb)Rh(Cp*)Cl]Cl throughout the catalytic cycles

In a first study Raman spectroscopy was employed to gain insight into the stability of 3 across the catalytic process. Therefore, the components in powder form for the photocatalytic system with catalyst **3** *i.e*. ([(dceb)Rh(Cp*)Cl]Cl ) were measured at a MultiRam Fourier-Transform Raman-Spectrometer (Bruker Corporation, Billerica, Massachusetts, United States of America) with a spectral resolution of 4 $cm^{-1}$ in the range 0 and 4000 $cm^{-1}$. The Raman excitation light at 1064 nm was provided by a Nd:YAG laser (Klastech DeniCAFC-LC-3/40, Dortmund, Germany). The laser power incident upon the sample plane was approximately 250 mW with 100 scans per sample. Background correction was performed using SNIP function (iterations:200, order:3, smoothing window: 1). Thereafter, the spectra were confined to the spectral range of 100 to 4000 $cm^{-1}$ and normalized using vector normalization to minimize variability in intensity of the samples.

In operando Raman spectroscopy of the **3** system was conducted using a NIR – excited fiber coupled Raman setup (RXN I, Kaiser Optical Systems, Ann Arbor, MI, USA). The sample was excited with a power of 250mW at the sample through a fiberoptic probe (InPhotonics, Norwood, MA, USA) which is equipped with band and long pass filters for cleanup and Rayleigh-rejection (cutoff long pass: ca.250$cm^{-1}$) with a focal spot width of about 50 µm which is coupled to a 785 nm single- frequency laser diode (Xtra II, Topica Photonics, Munich, Germany). This system disperses the scattered Raman light passing through a holographic grating and then detected via a multi-row, thermoelectrically cooled ($T_{OP}$ = -60 °C) CCD (Andor, Belfast, UK) resulting in spectral resolution around 4 $cm^{-1}$. The system was white light calibrated using a white light source prior to the measurements.

The **3** system was prepared in a glovebox under argon atmosphere as stated in the procedure and was conducted in a gas tight UV-grade quartz cuvette (Hellma Analytics, Müllheim, Germany) and data from 250 to 3500 $cm^{-1}$, was recorded by the system. To improve the signal to noise ratio six spectra were recorded and averaged for an integration time of 10 seconds per spectra. In operando studies were conducted under irradiation for the charging of the system using a LED source with a 455 nm (M455L4, Thorlabs Inc., Newton, NJ, USA). The LED power was set at 45 mW and was measured using an optical power meter (PM100D/S130VC, Thorlabs Inc. Newton, NJ, USA). The pre-processing and analysis of the data was done on the RAMANMETRIX

software Version 0.6.5-test1 (https://ramanmetrix.eu/, (accessed on 3 July 2024)). The spectra were confined to a wavenumber range of 200 to 2500cm$^{-1}$, background corrected using SNIP, smoothing window of 1, 40 iterations, and baseline smooth window of 11, and normalized using vector normalization of the area under the curve. The different components were measured, both each one individually and in an upscaled catalytic mixture (see Supplementary Fig. 15). It can be seen, that beyond the bipyridine vibrations, that are shared with the photosensitizer, the C=O double bond vibration of the ester can be observed as well in the individual measurement. In a catalytic mixture, the dominating vibrations are associated with the sacrificial donor and the polymer, due to the differences in concentration (1:5 for **3**:polymer, 1:2000 for **3**:TEA) and the nature of the vibronic signals. Due to the relatively low concentration of the catalyst in the catalytic mixture the identification of the carboxylic ester moiety within the rhodium catalyst was not possible. Nevertheless, in operando nonresonant FT-Raman studies were helpful to enable a reliable band assignment of the resonance Raman modes of the catalyst.

In further studies the catalytically active Rh(I)-species was investigated. Based on the previous results, this was done in absence of the polymer. Also to obtain the Rh(I)-species, a chemical reduction process was chosen, in order to avoid obstruction by either the photosensitizer or the sacrificial donor. Additionally, this species possesses an absorption feature, which allows for the use of resonance Raman (rR), enhancing sensitivity by a factor of $10^6$.

Resonance Raman measurements of intermediate states of the hydrogen evolution catalyst **3**, *i.e.* [(dceb)Rh(Cp*)Cl]Cl, were carried out using an excitation wavelength of 643 nm (i.e. within the reduction-induced absorption band arising from overlapping $^1$MLCT and $\pi^* \rightarrow \pi^*$ bands[7,8] (partially reduced bpy ligand in the $Rh^I$ state) of **3**, see Supplementary Fig. 16) of a diode-pumped solid-state laser (CrystaLaser, USA). The laser power at the sample was reduced to approximately 12 mW to minimize photodegradation of the $Rh^I$ chromophore. Raman signals were collected using an IsoPlane 160 spectrometer (Princeton Instruments, USA), with a 30 µm entrance slit and grating with 1200 grooves/mm. The spectrometer is equipped with a thermoelectrically cooled CCD camera (PIXIS eXcelon, Princeton Instruments, USA) featuring a resolution of 1340 x 100 pixels. The $H_2O$ spectral band at 1633 cm$^{-1}$ was utilized as a reference for normalizing both intensities and wavenumbers. For spectral

analysis, rR spectra were background-corrected, and the solvent spectrum was subtracted.

A series of aqueous solutions of 300 µM **3** was prepared in an Ar-filled glovebox (starting volume of each sample was 1 mL). These samples were then acidified to pH 2 employing 2.8 M HCl, before neutralizing it to pH 7 with 2.2 M NaOH, representing one catalysis cycle. By repeating these acidification and neutralization steps (two times for sample 2, three times for sample 3, etc.), samples matching the amount of pH swings of each of the four catalysis cycles were generated. Before removing these samples from the Ar-filled glovebox, an aqueous sodium formate solution was added, yielding a concentration of 20 mM formate in each of the sample solutions. The cuvettes were heated to 50 °C for about one hour in a water bath, to initiate Rh(I) formation via a reaction sequence of ß-hydride elimination/$CO_2$ evolution and deprotonation ($pK_a$ of the rhodium hydride species is 3.8, see Supplementary Fig. 9).[6] As the broad $^1$MLCT band of the reduced Rh complex (see Supplementary Fig. 9) strongly absorbs at the chosen excitation laser wavelength, the so-formed rRaman-active Rh(I) species were studied as representative samples of **3** / its degradation products in real catalytic cycles one to four.

Upon reducing the Rh(III) catalyst (**3**) using sodium formate, the formed Rh(I) species was investigated using resonance Raman spectroscopy at 643 nm. At this excitation wavelength only the catalytically competent intermediate, *i.e.*, the Rh(I) complex is absorbing. While we observed the Raman band of the carbonyl function at 1694 $cm^{-1}$ for the initial Rh(I) species, indicating an intact ester functional group at the bipyridine chromophore, this band vanishes after one or more exposure steps to low pH values. This is most likely because the functional group is hydrolyzed. While the C=O double bond is initially detected with an intact ester functional group, when the ethoxy group is cleaved at lower pH, the charges become delocalized, and the carbonyl vibration disappears. In addition, since the π electrons of the carbonyl group are also conjugated to the bipyridine chromophore, the change in the ester functional group also impacts the intensity ratios of the bipyridine bands, as can be seen when comparing the spectra of Rh(I)-0 (*i.e.* **3** exposed to no pH swing) with Rh(I)-1 to -4 (*i.e.*, **3** exposed to 1 to 4 pH swings; see Supplementary Fig. 17).

## 2. Figures and Tables

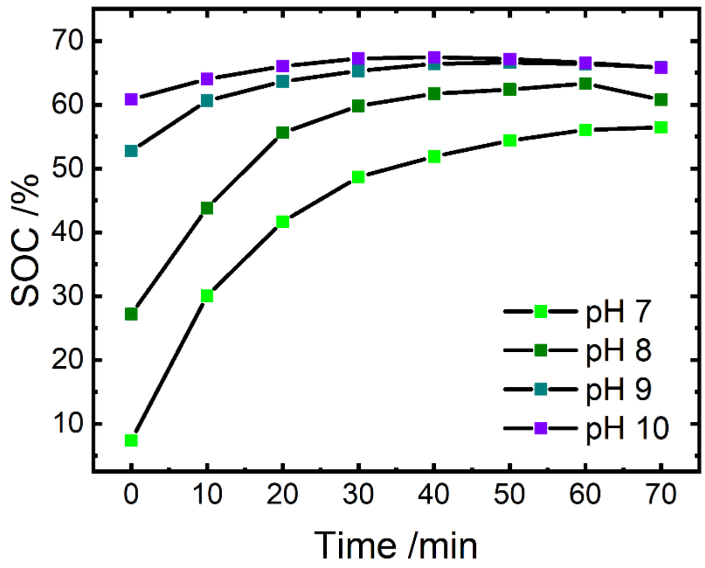


**Supplementary Fig. 1.** Degree of charging of the polymer (450 µM with respect to methyl viologen units) in an aqueous solution containing $[Ru(tbbpy)_3]Cl_2$ (25 µM), 0.18 M TEA and 0.15 M $NaH_2PO_4$ as well as the respective amount of NaOH (4 M) to reach pH 8, 9 and 10, tracked over 70 minutes of irradiation. SOC refers to state of charging.

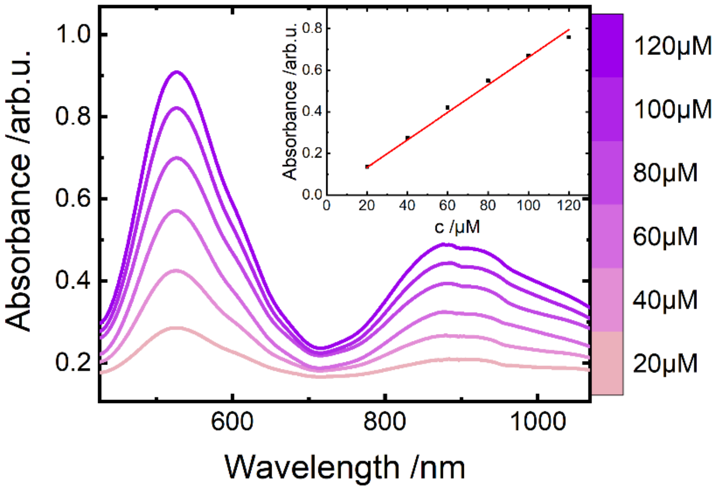


**Supplementary Fig. 2.** UV/vis absorption spectra of different amounts of the fully reduced polymer using $Na_2S_2O_4$ as reductant and the resulting linear regression (inset).

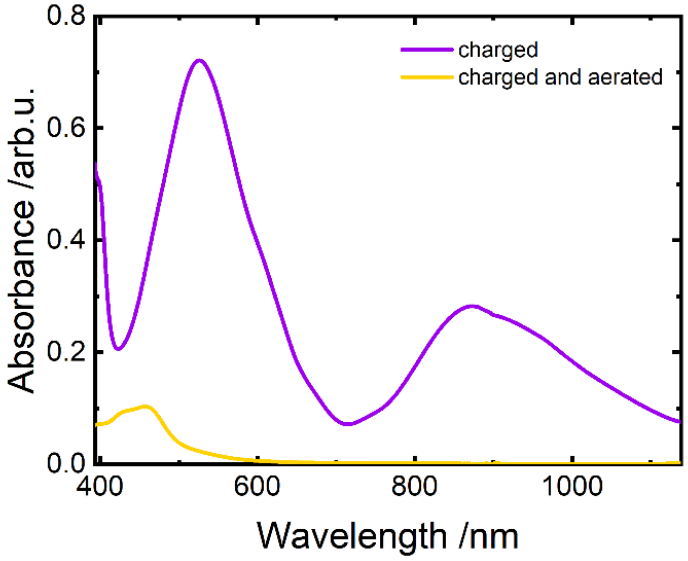


**Supplementary Fig. 3.** UV/vis absorption spectra of the photocatalytically charged copolymer (225 µM with respect to methyl viologen monomer content) using $[Ru(tbbpy)_3]Cl_2$ (12.5 µM) in water containing 0.09 M TEA and 0.075 M $NaH_2PO_4$ before and after aeration.

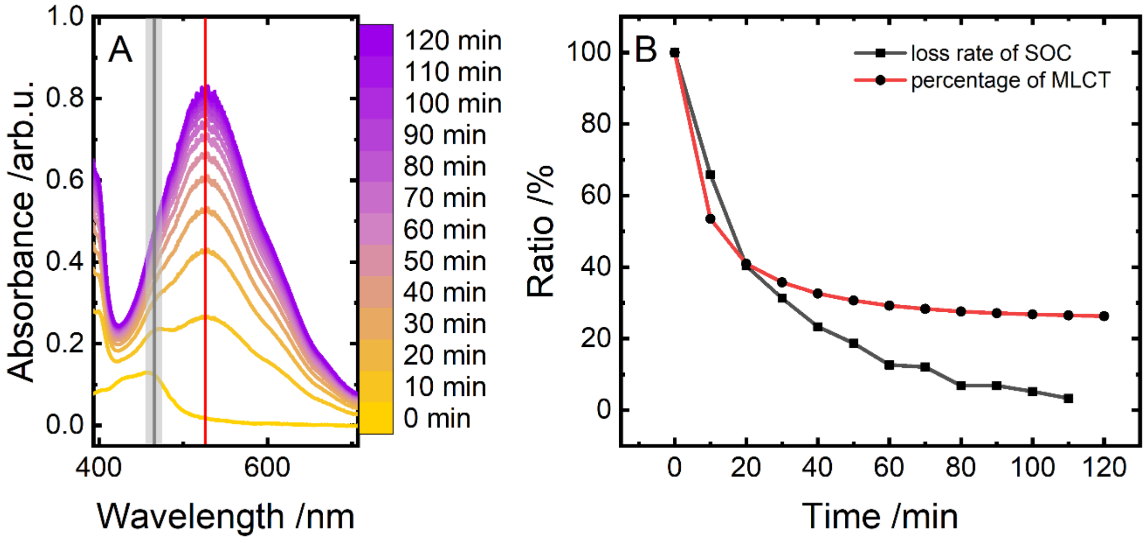


**Supplementary Fig. 4.** Relative SOC rate with respect to initial charge interval (red) and percentage of the MLCT absorption of the photosensitizer (grey) at the irradiation wavelength plotted against time (B) and an extract of the respective absorption profile with the respective cross-sections of the irradiation window (grey) and the SOC determining peak (red) marked (A). Experimental conditions are 225 µM with respect to methyl viologen monomer content, $[Ru(tbbpy)_3]Cl_2$ (12.5 µM) in water containing 0.09 M TEA and 0.075 M $NaH_2PO_4$.

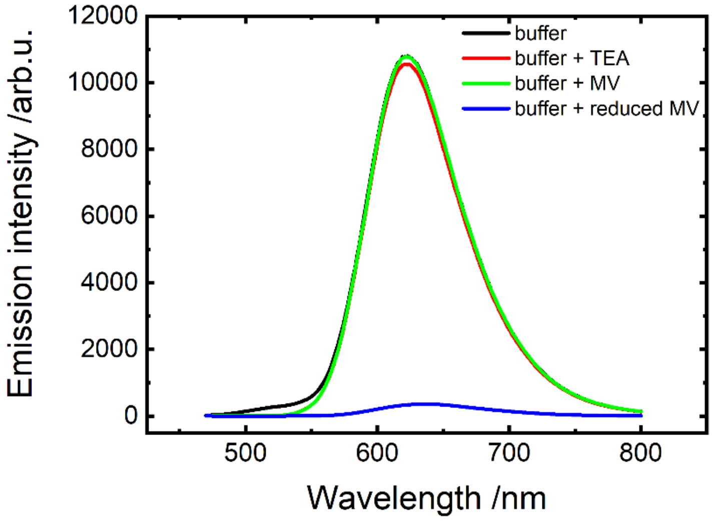


**Supplementary Fig. 5.** Emission spectra of $[Ru(tbbpy)_3]Cl_2$ (25 µM) containing 0.15 M $NaH_2PO_4$ (black), plus either 0.18 M TEA (red), the polymer (450 µM with respect to methyl viologen units) (green) or the chemically reduced polymer (450 µM with 60 eq. $Na_2S_2O_4$) all normalized by the absorption intensity at the excitation wavelength of 460 nm.

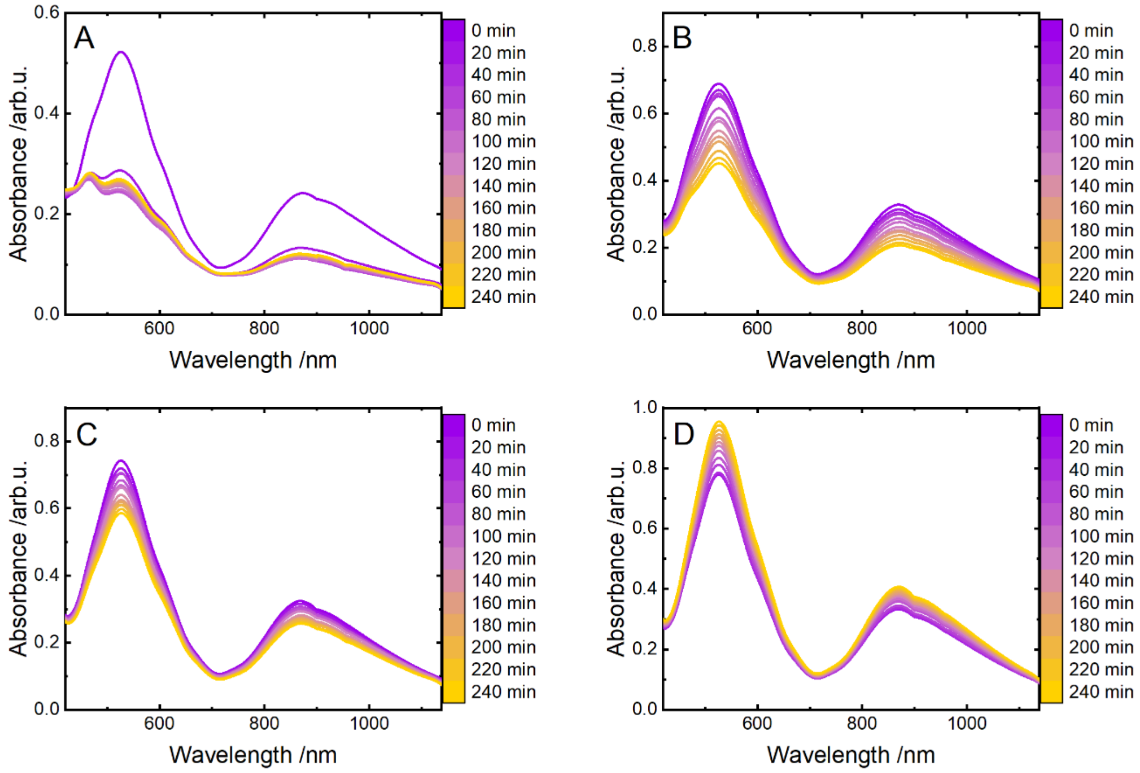


**Supplementary Fig. 6.** UV/vis spectroscopic changes of photochemically reduced polymer (450 µM with respect to methyl viologen content, 1 h of irradiation), using $[Ru(tbbpy)_3]Cl_2$ (25 µM) in water containing 0.18 M TEA and 0.15 M $NaH_2PO_4$ upon addition of 20 mol% with regard to MV-units of $K_2PtCl_4$ (A), Pt-NPs (B), [(dceb)Rh(Cp*)Cl]Cl (C) or [(tbbpy)Rh(Cp*)Cl]Cl (D).

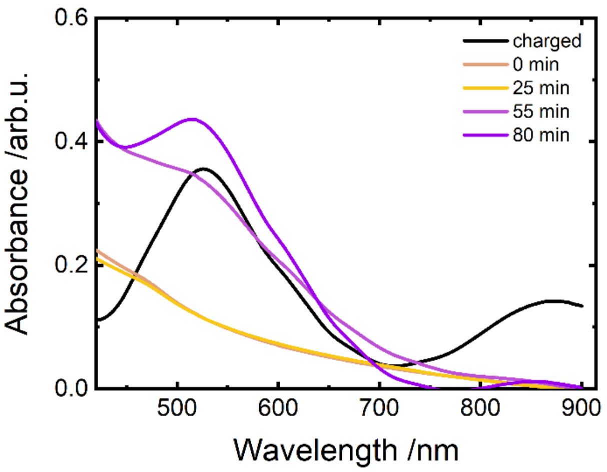


**Supplementary Fig. 7.** UV/vis spectra of the charged polymer (200 µM) using [Ru(tbbpy)$_3$]Cl$_2$ (5 µM) in an aqueous solution containing 0.2 M TEA and 0.16 M NaH$_2$PO$_4$, plus the spectra following this charging after adding **1** and further irradiation for the given time.

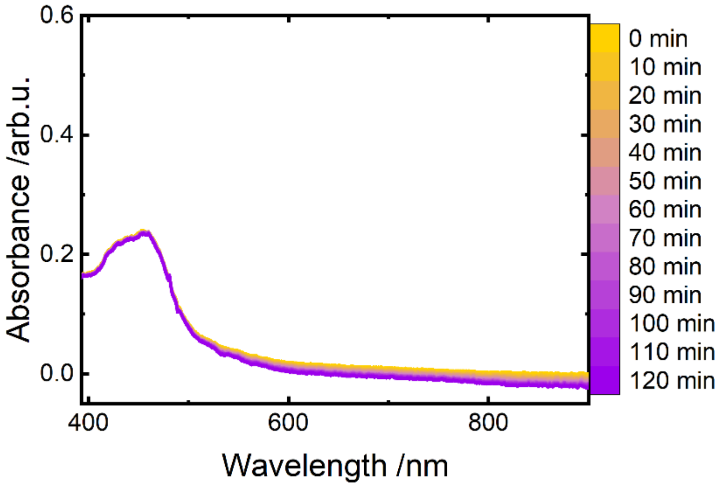


**Supplementary Fig. 8.** UV/vis absorption spectra of the absent reduction of the copolymer (225 µM with respect to methyl viologen monomer content) using [Ru(tbbpy)$_3$]Cl$_2$ (12.5 µM) in water containing 0.09 M TEA and 0.075 M NaH$_2$PO$_4$ after the addition of hydrogen.

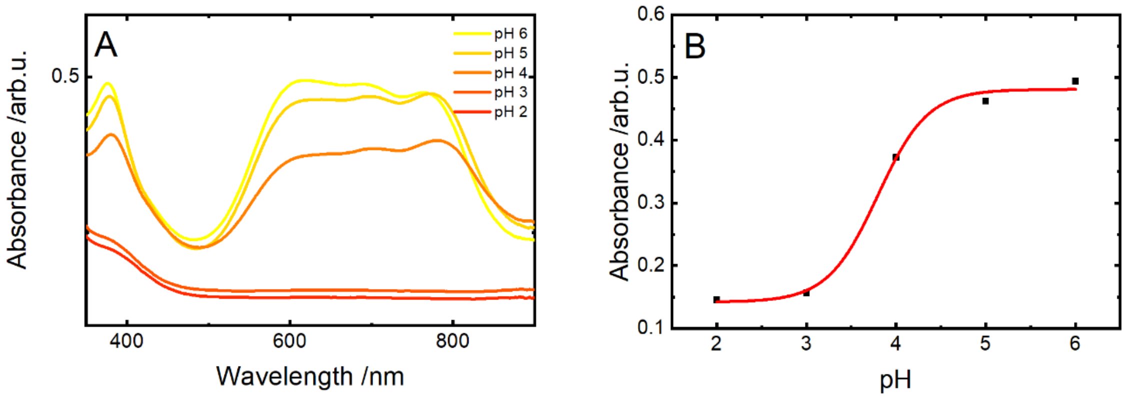


**Supplementary Fig. 9.** Absorption spectra at different pH values following formate-induced reduction of [(dceb)Rh(Cp*)Cl]Cl (A). Using a sigmoidal fit of the absorption at 616 nm (B), a $pK_a$ value of 3.8 for the two electron reduced and protonated catalyst species was determined, highlighting the necessity for sample acidification in combination with catalyst addition for driving $H_2$ evolution with [(dceb)Rh(Cp*)Cl]Cl using the electrons stored on the photochemically reduced polymer.

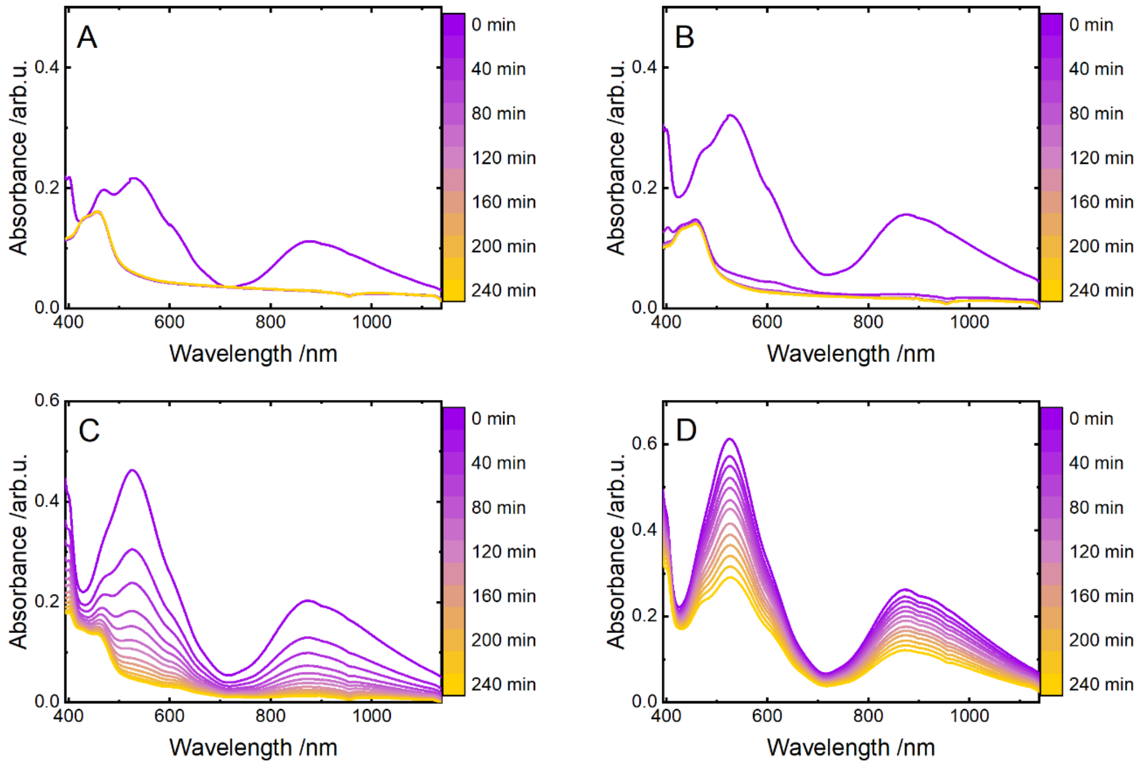


**Supplementary Fig. 10.** UV/vis spectroscopic changes of photochemically reduced polymer (450 µM with respect to methyl viologen content, 1 h of irradiation), using $[Ru(tbbpy)_3]Cl_2$ (25 µM) in water containing 0.18 M TEA and 0.15 M $NaH_2PO_4$ upon addition of 20 mol% $K_2[PtCl_4]$ (A), Pt(0)-NPs (B), [(dceb)Rh(Cp*)Cl]Cl (C) or [(tbbpy)Rh(Cp*)Cl]Cl (D) following acidification of the sample to pH 2 using HCl (2 M).

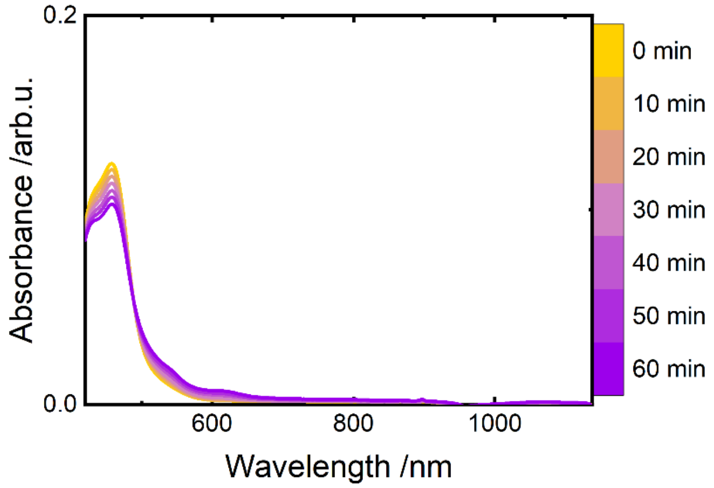


**Supplementary Fig. 11.** UV/vis spectroscopic changes of polymer (450 µM with respect to methyl viologen content, 1 h of irradiation), using $[Ru(tbbpy)_3]Cl_2$ (25 µM) in water containing 0.18 M TEA and 0.15 M $NaH_2PO_4$ at pH 6.

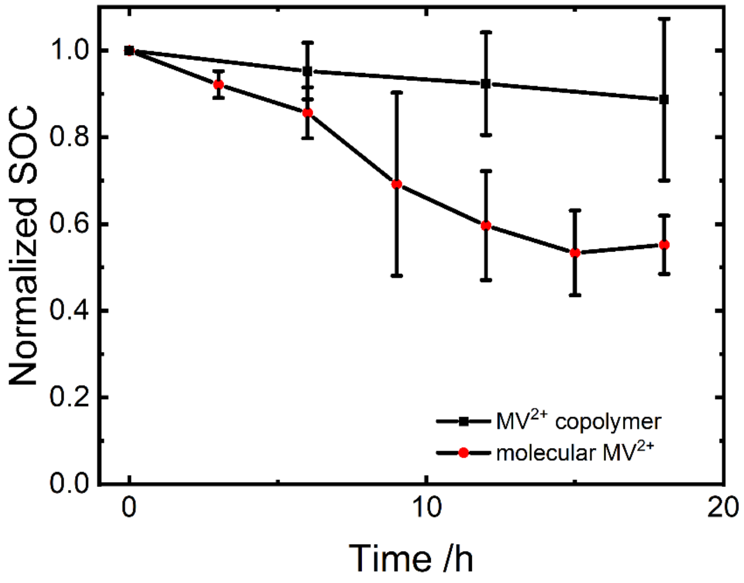


**Supplementary Fig. 12.** Normalized absorption profile of charged samples of polymeric $MV^{2+}$ (black) and molecular $MV^{2+}$ (red) within the first 15 h after charging. SOC refers to state of charging. Error bars represent standard deviation of n = 3 independent measurements.

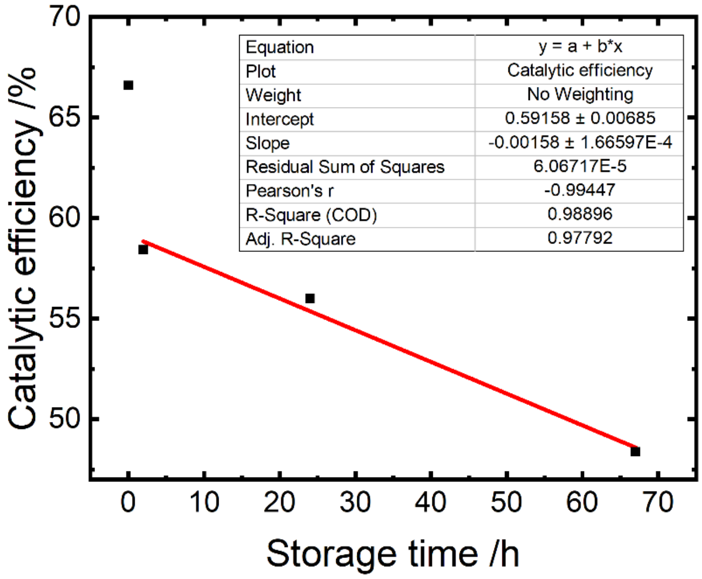


**Supplementary Fig. 13.** Catalytic efficiency depending on the time the charged polymer sample was stored before initiating catalysis together with a respective plot of the linear part of the decay.

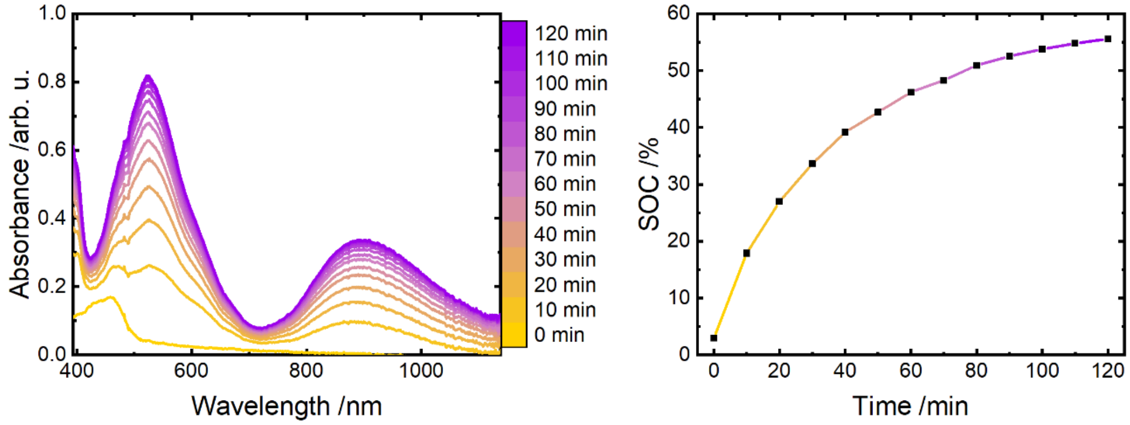


**Supplementary Fig. 14.** UV/vis absorption spectra during photocatalytic charging of the copolymer (225 µM with respect to methyl viologen monomer content) using [Ru(tbbpy)$_3$]$Cl_2$ (12.5 µM) in water containing 0.09 M TEA and 0.075 M $NaH_2PO_4$ in the presence of 355 mM NaCl. SOC refers to state of charging.

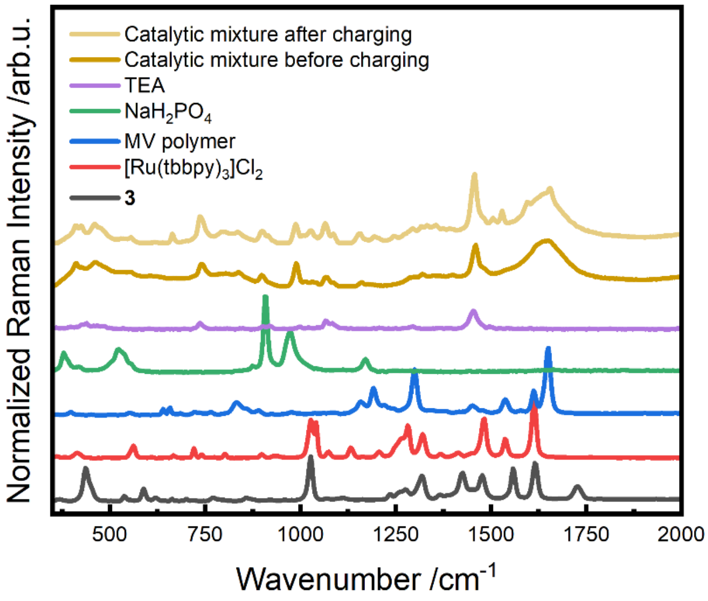


**Supplementary Fig. 15.** Experimental Raman spectra of **3**, $[Ru(tbbpy)_3]Cl_2$, MV-polymer, $NaH_2PO_4$ and triethylamine individually in an upscaled catalytic mixture before and after irradiation.

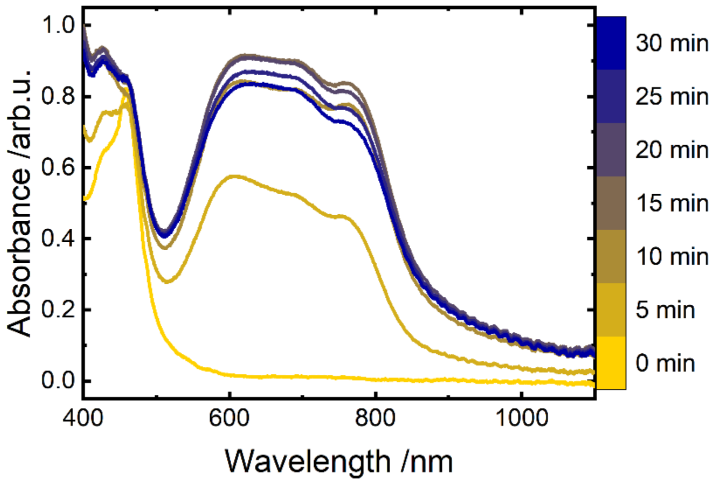


**Supplementary Fig. 16.** UV/vis spectroscopic changes of photochemically reduced **3** (150 µM) over 30 min of irradiation, using $[Ru(tbbpy)_3]Cl_2$ (42 µM) in water containing 0.09 M TEA and 0.075 M $NaH_2PO_4$.

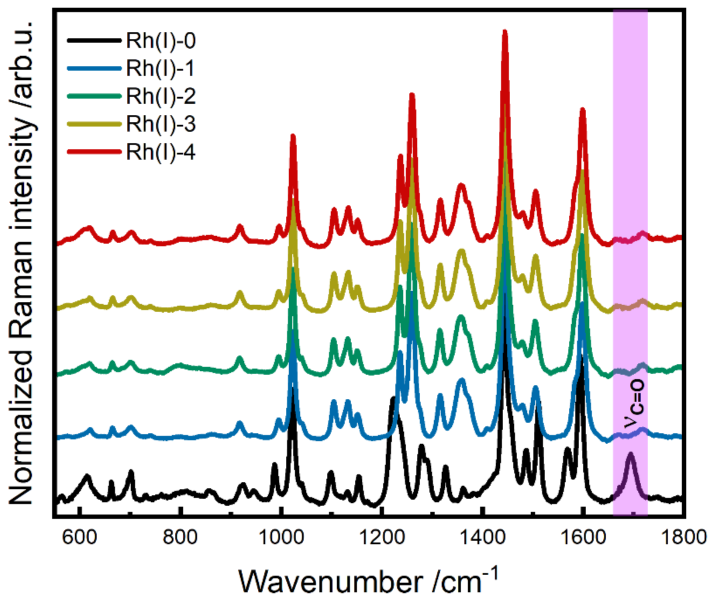


**Supplementary Fig. 17.** Experimental resonance Raman spectra of chemically reduced **3** in aqueous solution after exposure to n acid/base pH swings (n = 0-4), excited at 643 nm.

**Supplementary Table 1.** Amount of $H_2$ generated (in nmol) after 2 h in absence of either catalyst (**2**) or HCl.

| | Sample 1 | Sample 2 | Sample 3 | Resulting average efficiency |
|---|---|---|---|---|
| No catalyst | 0 | 0 | - | 0% |
| No HCl | 0.470 | 0.474 | 0.460 | 4% |

**Supplementary Table 2.** Amount of $H_2$ generated (in nmol) by Pt(0) after 2 h and after 3 h employing either molecular or polymeric $MV^{2+}$.

| | Sample 1 | | Sample 2 | | Sample 3 | |
|---|---|---|---|---|---|---|
| | 2 h | 3 h | 2 h | 3 h | 2 h | 3 h |
| Molecular $MV^{2+}$ | 2.713 | 1.828 | 2.03 | 1.526 | 2.132 | 1.483 |
| $MV^{2+}$ copolymer | 6.097 | 5.312 | 5.964 | 5.738 | 6.271 | 5.531 |

**Supplementary Table 3.** Average TON for on-demand $H_2$ evolution per MV repetition unit ($TON_{max}$ = 0.5 for 100 % charging of the polymer; based on n = 3 independent measurements).

| | 0.5 h | 1 h | 2 h | 4 h | 5 h | 23 h |
|---|---|---|---|---|---|---|
| $K_2[PtCl_4]$ (**1**) | 0.020 | 0.026 | 0.024 | 0.025 | 0.029 | 0.026 |
| Pt(0) (**2**) | 0.188 | 0.206 | 0.224 | 0.205 | 0.198 | 0.187 |
| [Rh(dceb)Cp*Cl]Cl (**3**) | 0.067 | 0.107 | 0.121 | 0.155 | 0.166 | 0.170 |
| [Rh(tbbpy)Cp*Cl]Cl (**4**) | 0 | 0.005 | 0.012 | 0.028 | 0.031 | 0.060 |

**Supplementary Table 4.** Average TON for on-demand hydrogen evolution per HEC ($TON_{max}$ = 2.5 for 100 % charging of the polymer due to catalyst amount of 20 mol%; based on n = 3 independent measurements).

| | 0.5 h | 1 h | 2 h | 4 h | 5 h | 23 h |
|---|---|---|---|---|---|---|
| $K_2[PtCl_4]$ (**1**) | 0.100 | 0.132 | 0.120 | 0.124 | 0.145 | 0.130 |
| Pt(0) (**2**) | 0.940 | 1.028 | 1.122 | 1.027 | 0.990 | 0.934 |
| [Rh(dceb)Cp*Cl]Cl (**3**) | 0.333 | 0.536 | 0.606 | 0.777 | 0.832 | 0.848 |
| [Rh(tbbpy)Cp*Cl]Cl (**4**) | 0 | 0.023 | 0.061 | 0.139 | 0.154 | 0.302 |

## 3. Calculations

3.1. Catalytic efficiency

The raw data from the GC was obtained as µmol hydrogen in 100 µL of extracted syringe volume. This measurement of the hydrogen content in the headspace of a sample enables the determination of the catalytic efficiencies according to the following calculations. First of all, the amount of hydrogen is multiplied the respective factor $X$ (ranging from 20.18 to 26.15 depending on sample volume) to account for the difference between headspace and sample volume (100 µL).

$$n_{sample} * X = n_{total}$$

This total amount of hydrogen ($n_{total}$) can then be divided by the amount of methyl viologen units present in the sample and thus yields $TON_{MV}$. Alternatively, the turnover number (TON) could be calculated with respect to the catalyst ($TON_{CAT}$) by division with the amount of catalyst in the sample.

$$n_{total} / n_{MV} = TON_{MV}$$

To account for the fact that two reduced protons are required to form one molecule of hydrogen this $TON_{MV}$ has to be doubled. This represents the efficiency of a theoretical system with all methyl viologen units being charged. Therefore, this theoretical efficiency is corrected by the respective degree of charging, yielding the catalytic efficiency.

$$(2 * TON_{MV}) / \textit{degree of charging} = \textit{catalytic efficiency}$$

Error margins in all cases were represented by the standard deviation, as square route of the averaged variance. The variance is in turn given by the difference between data and average to the power of two.

3.2. Charge per mass

With the Avogadro constant $N_A$ and the molar mass $M$ (571.02 g/mol) of the average repeating unit, bearing one viologen moiety and one solubility enhancing styrene-ammonium group, one can determine the number of electrons $n_e$ in 1 g of copolymer. Since this would account for 100% of the viologen units being charged with exactly one

electron. This value has to be multiplied by the SOC of 82%, to consider the percentage of actually charged units.

$$n_e = \frac{\frac{1\,g}{M}}{N_A} * 0.82$$

This total number of electrons in one gram of polymer is then multiplied by the elementary charge $e$ to yield the storage capacity of the copolymer.

$$Storage\ capacity = n_e * e$$

<u>References:</u>


1. Mengele, A. K. & Rau, S. The Metallic Traveler: Formate- And Photoinduced Regioselective Phenanthroline Deuterations via Reductively Activated RhCp∗ Centers. *Organometallics* **39**, 2739–2748 (2020).
2. Todd, A. M., Swinburne, A. N., Goeta, A. E. & Steed, J. W. Anion receptor coordination tripods capped by [9]ane-S3. *New Journal of Chemistry* **37**, 89–96 (2013).
3. Janoschka, T. *et al.* An aqueous, polymer-based redox-flow battery using non-corrosive, safe, and low-cost materials. *Nature* **527**, 78–81 (2015).
4. Geraskina, M. R., Dutton, A. S., Juetten, M. J., Wood, S. A. & Winter, A. H. The Viologen Cation Radical Pimer: A Case of Dispersion-Driven Bonding. *Angewandte Chemie International Edition* **56**, 9435–9439 (2017).
5. Pfeffer, M. G. *et al.* Palladium versus Platinum: The Metal in the Catalytic Center of a Molecular Photocatalyst Determines the Mechanism of the Hydrogen Production with Visible Light. *Angewandte Chemie International Edition* **54**, 5044–5048 (2015).
6. van Esch, J. H., Hoffmann, M. A. M. & Nolte, R. J. M. Reduction of Nicotinamides, Flavins, and Manganese Porphyrins by Formate, Catalyzed by Membrane-Bound Rhodium Complexes. *Journal of Organic Chemistry* **60**, 1599–1610 (1995).
7. Kaim, W. & Fiedler, J. Spectroelectrochemistry: the best of two worlds. *Chem. Soc. Rev.* **38**, 3373 (2009).
8. Cameron, L. A., Ziller, J. W. & Heyduk, A. F. Near-IR absorbing donor–acceptor ligand-to-ligand charge-transfer complexes of nickel( II ). *Chem. Sci.* **7**, 1807–1814 (2016).
